# Factors impacting implementation of nutrition and physical activity policies in rural schools

**DOI:** 10.1186/s12889-023-15176-y

**Published:** 2023-02-10

**Authors:** Caryn Ausenhus, Joshua M. Gold, Cynthia K. Perry, Andrea Kozak, Monica L. Wang, Sou Hyun Jang, Judy Leong, Edgar Rodriguez, Catherine Duggan, Linda K. Ko

**Affiliations:** 1 Public Health Sciences Division, Fred Hutchinson Cancer Center, 1100 Fairview Ave. N, M3-B232, Seattle, WA 98109 USA; 2 Department of Health Systems and Population Health, University of Washington, Seattle, USA; 3 Oregon Health & Science University, School of Nursing, 3455 SW US Veterans Hospital Rd., SN-ADM, Portland, OR 97239 USA; 4 Department of Psychology, Oakland University, 654 Pioneer Drive, Rochester, MI 48309 USA; 5 Department of Community Health Sciences, School of Public Health, Boston University, 715 Albany Street, Boston, MA 02118 USA; 6 Department of Sociology, Korea University, 145 Anam-Ro, Anam-Dong, Seongbuk-Gu, Seoul, South Korea; 7 U.S. Department of Veterans Affairs, VA Puget Sound Healthcare System, 1660 S. Columbian Way, Seattle, WA 98108 USA

**Keywords:** Childhood obesity, Rural school, Nutrition, Physical activity, Latino students, Implementation

## Abstract

**Background:**

Rural Latino children have higher rates of obesity compared to non-Latino Whites. Schools are in a unique position to address rural childhood obesity through policies. While evidence exists on factors that promote or impede school-based physical activity (PA) and nutrition policies, only a fraction has been in rural communities. This study seeks to understand 1) the knowledge and perceptions of school nutrition and PA policies and 2) barriers and facilitators to their implementation among rural school stakeholders from Washington State.

**Methods:**

We conducted 20 semi-structured, in-depth interviews with school stakeholders (e.g., principals and school nutrition directors) from four K–12 school districts in the Lower Yakima Valley of Eastern Washington State. Thematic analysis was conducted using inductive, constant comparison approach to identify themes around knowledge and perceptions of policies and barriers and facilitators of policy implementation.

**Results:**

Three main themes were identified: perceptions and knowledge of school PA and nutrition policies, barriers to policy implementation, and facilitators of policy implementation. The majority of stakeholders were supportive of school-based policies promoting PA and a healthy diet, even when lacking a specific understanding of these policies. Four subthemes were identified as barriers to policy implementation: viewing PA as a low priority, misuse of recess time, funding constraints, and lack of strong leadership. Facilitators of implementation included strong leadership at the district level, creating healthy norms through school-community linkages and pooling community resources to improve nutrition and PA among children.

**Conclusions:**

Schools provide a unique setting to promote healthy diet and PA behaviors among children and their families. Study findings show that while knowledge of specific nutrition and PA policies may vary, support for such policies were high among rural stakeholders. Study findings can inform policy development and support strategies for policy implementation in rural settings. Future studies may want to examine whether implementation of strategies addressing the barriers and enhancing facilitators lead to success in rural school settings.

**Supplementary Information:**

The online version contains supplementary material available at 10.1186/s12889-023-15176-y.

## Background

One in five school-age children in the United States (US) is obese [1]. All regions of the US are affected by this epidemic; however, children living in rural areas are 26% more likely to be obese than their urban counterparts [2]. Major determinants of disparity include limited opportunity for physical activity (PA) [3], lack of physical infrastructure for PA [4], lack of access to healthy food [5] or community resources [6], and higher rates of food insecurity [7]. Racial disparities in obesity also exist among rural populations; rural Latino children have higher rates of obesity compared to non-Latino Whites [8].

Due to the complex and interdependent nature of these determinants, researchers have adopted a systems-oriented perspective to address the multiple factors and levels that contribute to childhood obesity [9]. Schools represent a unique nexus point within a community and have the potential to create change on individual, family, and community levels [10]. Given the general lack of healthy food and PA opportunities in rural communities [11], schools are in a unique position to address the rural childhood obesity crisis [12, 13] by providing students with healthier nutritional options and structured time for PA. Federal school nutrition programs such as the US Department of Agriculture’s School Breakfast Program (established in 1966), the National School Lunch Program (2004), and the Healthy Hunger-Free Kids Act (2010) represent an opportunity to tackle obesity through the provision of healthy food and education programs. The Centers for Disease Control and Prevention (CDC) recommends that schools implement nutrition policies that provide students with nutritious and appealing foods, drinking water and beverages, and accurate messaging about good nutrition and healthy eating through food and beverage marketing, staff role modeling, and healthy eating learning opportunities [14]. Similarly, a combination of school-based PA policies, such as requiring physical education (PE) courses, daily recess, and in-class PA breaks, can help rural children achieve 60 min of daily PA as recommended by the CDC [15].

A recent systematic review of mostly urban schools examining the effect of nutrition policies on children’s dietary behaviors showed that direct provision of healthy foods, competitive food/beverage standards, and school meal standards improved fruit and vegetable intake and reduced sugar-sweetened intake [16]. Similarly, a large study using time series design found that school nutrition policies contributed to containing the upward trend in childhood overweight/obesity among 5^th^-and 7^th^graders in California public schools and narrowing disparities gap specially among non-Hispanic Black and Latino children [17]. Interestingly, the effect of PA policies was noted as inconclusive, with inconsistent association between state-level PA policies and school-day PA practice, and ultimately school children’s behavior, highlighting the complexity of translating state-level policies into school-level practice [18].

Despite the benefits of school-based nutrition and PA policies on child health and wellbeing, a scoping review of mostly urban schools, showed that schools face several barriers to both implementing and adhering to such policies. For example, low levels of macro-level (i.e., district and state-level) support, high cost of healthy foods, misalignment of nutrition and academic priorities (e.g., administrative emphasis on subjects other than nutrition), lack of shared responsibility among stakeholders, and overlooked community characteristics have been identified as barriers to nutrition policy implementation [19]. For PA policies, a systematic review showed that environmental context and resources, social influences, and the low priority of PA compared to other academic subjects, as prominent factors affecting PA policy implementation in schools [20].

While previous studies offer valuable insight into the factors that promote or impede school-based PA and nutrition policy implementation [19, 20], only a fraction were in rural communities. Furthermore, these studies often evaluate nutrition and PA policies as independent rather than as intertwined, dynamic policies that can influence each other. Examining implementation of nutrition and PA policies in rural schools is critical; compared to urbanized schools, rural schools have inadequate physical infrastructure [4], reduced funding [21], higher teacher turnover [22], and lower school and community resources [6], while students that they teach experience higher levels of obesity [8], higher levels of food insecurity [1], and come from low social-economic status. Understanding factors that hinder and facilitate implementation of nutrition and PA policies can inform policy development and support strategies for policy implementation in rural schools. This study seeks to understand 1) the knowledge and perceptions of school nutrition and PA policies and 2) barriers and facilitators to their implementation among rural schools in Washington State.

## Methods

### Procedure

This is a qualitative research study using semi-structured, in-depth interviews with school stakeholders to understand their knowledge and perceptions of PA and nutrition policies at rural schools and the factors that influence policy implementation. This study was part of a larger community-academic partnership study—The Collaboration for Healthy Communities—that focused on capacity and infrastructure building through community engagement, community needs assessment, and a community-focused pilot intervention study; a community advisory board advised the research team throughout the study [23–25].

This study took place in four rural communities in the Lower Yakima Valley of Eastern Washington State. The Lower Yakima Valley includes many small agricultural communities and has a population of approximately 100,000. Approximately 67% of the population is Hispanic [26]. Many Latinos work in the agricultural industry, including farm work, packing warehouses, and other related work [27]. Participants came from four school districts from K-12 with approximately 400 students in each school. The school district size ranged from small (3 schools) to middle (5 schools) to large (8 schools) for rural settings. All students were enrolled in the free (eligible households have family incomes below 130 percent of the poverty line) or reduced-price (eligible households have family incomes below 185 percent of poverty line) lunch program [28].

### Participants

Eligibility criteria included being 18 years old or older and employed at one of the participating school districts. The community advisory board members connected the study team with superintendents from the four school districts. The superintendents provided names and contact information of those who may be interested in participating in the interviews. This list was augmented with names found in the district and school websites, and contact details given by those who completed the interview. Data saturation was reached after 20 school representatives across four school districts participated in the study. Study participants were members of the school board, school leadership, teaching staff (classroom and PE teachers), and nutrition program (Table 1). Bilingual/bicultural community health workers contacted potential participants by telephone to explain the study and schedule an in-person interview. All participants contacted agreed to participate in the study.

**Table 1:** Demographic Characteristics of the Participants (n=19)^a^

Variables	n (%)
Age, Mean (SD)	47.6 (13.3)
Ethnicity	
Hispanic	9 (47.4)
Non-Hispanic	10 (52.6)
Race	
White	9 (47.4)
Hispanic	9 (47.4)
American Indian/Native American	1 (5.3)
Gender	
Male	8 (42.1)
Female	11 (57.9)
Marital Status	
Single	4 (21.1)
Married or living in a marriage-like relationship	15 (79.0)
Annual Household Income	
Less than $15,000	1 (5.0)
$15,000 to less than $35,000	5 (26.0)
$35,000 to less than $50,000	4 (21.0)
$50,000 or more	9 (47.0)
Employment Status	
Full time	16 (84.2)
Part time	3 (15.8)
Employment length, Mean (SD)	10.9 (10.5)
Years of education, Mean (SD)	15.9 (3.1)
Position at school/district^b^	
Teacher	2 (11.0)
Food Services and Transportation Director	2 (11.0)
Administration (Superintendent, Program directors)	4 (21.0)
Nurse	2 (11.0)
Specialist and counselor	2 (11.0)
School board member	1 (5.0)
Other	2 (11.0)
Health Insurance	
Private health insurance	18 (94.7)
None	1 (5.3)
Health Status	
Excellent	2 (10.5)
Very good	13 (68.4)
Good	4 (21.1)

^a^ One participant did not complete the demographic survey

^b^ Two participants did not provide their position at school/district

Interviews took place at a community location (school, community center, and local study office) convenient to the participant in-person. Interviews lasted 1–1.5 h, were audio-recorded, and transcribed verbatim for data analysis. Participants gave written informed consent prior to participation, completed an interviewer-administered paper-based demographic survey, and were compensated with a gift card of $25 for their time. One participant did not complete the demographic survey. Data were collected from January to March 2014. This study was approved by the Institutional Review Board of the Fred Hutchinson Cancer Research Center.

## Instrumentation

Interview questions were designed to understand participants’ knowledge and perceptions of state- and national-level school PA and nutrition policies, factors that hinder or facilitate implementation of those policies, and how schools might improve policy implementation (see Appendix for the Interview Guide). Examples of questions include, “Can you tell me what policies you have in your school around physical education, what makes it difficult for schools to implement policies around physical education, and what would make it easier to implement policies for physical education?” We first asked questions related to PA policies and then asked questions regarding nutrition policies. Prior to the interviews, we received input from community health workers and the community advisory board on the questions to assess community relevance and revise the guide as appropriate.

## Data analysis

Descriptive analyses generated frequencies for categorical variables and means for continuous variables for the demographic survey data. Four researchers (LKK, CA, SHJ, and JL) used an inductive, constant comparison approach [29] in which concepts were identified and themes derived from the interview data. Using an iterative process, researchers met biweekly to refine the codebook (i.e., adding, removing, and revising codes as needed), address inter-rater agreement, and compare new codes with existing codes. Researchers used consensus to resolve coding discrepancies. The data were organized using ATLAS.ti, version 7 (Scientific Software Development, Berlin, Germany, 2013).

## Results

**Fig. 1:**
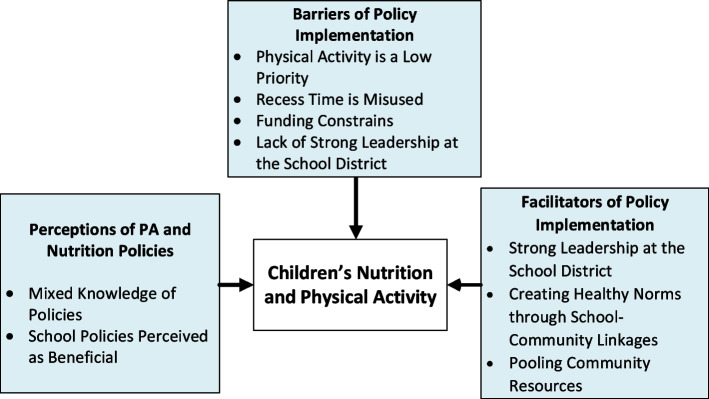
Factors impacting implementation of nutrition and physical activity policies

Participants’ mean (standard deviation) age was 47.6 (13.3) years. About half of the participants were Latino (47.4%), female (57.9%), and reported an income of $50,000 or more (47%). Most were employed full time (84.2%), married (79%), and insured (94.7%). The mean length of employment was 10.9 (10.5) years. Of the 20 participants who completed the interviews, four were superintendents or program directors, two were teachers, two were nutrition services and transportation director, two nurses, two specialists, and one school board member. Two participants did not disclose their position (Table 1). The school districts ranged from small, medium, to large district. The school district that was small in size had three schools (one elementary, one middle, and one high school). Two districts were middle in size. One of the two had five schools (three elementary, one middle, and one high school); the second district had six schools (four elementary, one middle, and one high school). The largest school district had eight schools (six elementary, one middle, and one high school). Figure 1 provides a pictorial overview of our results. The three shaded boxes are themes and subthemes that were identified as contributing to nutrition and PA among children. Below we discuss participants’ perceptions of school policies for nutrition and PA, followed by barriers and facilitators of implementation.

### Perceptions and knowledge of school PA and nutrition policies

#### Mixed knowledge of policies

Many participants were confident their schools had policies surrounding PA. However, when asked to describe details of their school’s specific PA policies, most either identified them incorrectly or were not able to state them. Only two participants were able to state the policy of 100 minutes of PE per week as noted in the Washington State learning standards [30]. When asked if their schools were meeting the standards of 100 minutes per week, many did not believe that was the case.

In contrast to PA, there was an overall perception that the schools were implementing recommended nutrition policies. Many participants were able to identify one or more nutrition policies, including a free/reduced-price lunch program, diverse healthy menu options for breakfast and lunch, a fresh fruit and vegetable program, and vending machine policies. Some mentioned the district's emphasis on providing more nutritious meals for students by reducing carbohydrates, serving smaller portion sizes, and implementing a salad and fruit bar. One participant summed up the different ways that schools served healthy meals.

*…We…tried to on [our] menus…offer…diverse menu options, each week…we might do chicken…a salad item one day or sandwich…to give them a more broad…spectrum of…what is actually out there to eat. And we also do a program, it’s called the ‘fresh fruits and vegetables’ program in that we provide a snack each afternoon for all the elementary kids and…broaden our horizons and to get them to try different items…different fruits and vegetables that they may not…get to try normally. (Participant# 12, Food Service Director)*

#### School policies perceived as beneficial

All the participants perceived the school PA and nutrition policies as beneficial for the children and their families. Many felt that school provided a platform of “tremendous impact” for shaping habits: children observe and learn from adult role models, like teachers, who are “motivated” to be physically active, and children “emulate” what they see. Many believed that the early education and prioritization of PA equips students with skills and habits that they can carry into adulthood. In addition to the benefits of offering free/reduced-price healthy meals to students, discussions centered around health education opportunities for kids. Some participants described ways teachers could incorporate the importance of healthy nutrition in their curriculum and introduce children to new fruits and vegetables.

Similar to PA policies, the same participant emphasized the importance of early nutrition education as it may shape the eating habits that continue into adulthood. Others mentioned that school plays a critical role in normalizing healthy food for children, and children could be a conduit for imparting the knowledge they learned about healthy food in school to their families.

*If they are eating something here [meaning a healthy meal] and then they can go home and say ‘Mom, no, I don’t want to eat [i.e. an unhealthy meal]…I want to eat what the school is giving me and that tastes good’…I think that if the school was able to…provide the kids with healthier foods, then the kid will say, ‘Well, this is normal, this is what it’s supposed to look like, this is what it’s supposed to taste like, and it’s supposed to be good for me and it’s OK to eat it.’ (Participant #6, Elementary School Counselor)*

### Barriers to policy implementation

#### Physical activity is a low priority

Many participants mentioned the challenges of implementing PA policies in schools as they were often not the school’s priority. A few participants noted the school’s intention to fully support implementation of PA policies. Yet, when resources for core curriculum such as reading, math, and science are limited, academic pressure takes priority over implementing PA policies, and PE time tends to be “borrowed” to supplement testing preparation.

*I think…a lot of the school are just really focused on the reading, math, science, because…the state is so hard on getting those test scores where they need to be per school district and if the school district is lacking in those areas, then…the physical education is there but it’s pushed to the side. (Participant #10, High School Administrator)*

Teachers were viewed as being caught in the middle of these competing priorities. Participants noted that teachers are most pressured to meet the academic expectations of the state but also understand the importance of children’s health and believe that should be their priority as well. Noting how these two expectations collide, a teacher shared their frustration in attempting to meet the expectation of the school and stay true to their sense of responsibility to the community.

*…We’re talking about academic achievement. We’re not necessarily talking about health achievement, health improvement in the schools because teachers—that’s not our job but…if we’re truly reflecting the community, that should be* *a part of our job. (Participant #4, Indian Education Specialist)*

While some participants believed schools could better enforce PA policies across school personnel, most indicated that this should be more than a local concern. State and national priorities around “school reforms” and “school improvement” must expand beyond “meeting the testing standards” and include PE.

#### Recess time is misused

While schools allocate time for recess, many participants reported that students were not being physically active during this designated time. Reasons included lack of school infrastructure for indoor PA, a tight school schedule, students choosing not to be active, and disciplinary methods, all of which contribute to sedentary behaviors. Starting with infrastructure, some participants noted that the lack of a separate gym and lunchroom space contribute to low levels of PA during recess time on “severe winter days.” Consequently, on these days, students are kept in their classroom during recess and asked to watch movies. A busy school schedule was also noted as a factor impacting recess time in that lunch and recess are often combined, and one of the two tends to suffer. Others noted that children are choosing to be less active at recess and instead elect to sit and talk with one another. Finally, taking time away from recess is often used as a disciplinary action, where children are kept off the playground or told to sit out as a result of their misbehavior. One participant shared concern about the misused recess time, limiting children’s ability to be more physically active.

*But we often see is that when kids get in trouble or there’s discipline issues, that’s the first thing taken away…[is] recess…. If it’s too cold outside, they take away that recess, so kids aren’t getting that access to the daily activity being able to go outside and run, which also helps kids focus because they’re getting that exercise. (Participant #15, School Board Member)*

#### Funding constraints

Many participants mentioned that constrained funding at the district level limited the school’s ability to offer students a wider variety of fresh foods and opportunities for PA. They mentioned that the district administration decides how to allocate state funds to the different school programs. Many times, funding for nutrition programs becomes insufficient, leaving only enough to purchase prepackaged meals, and some participants stated that they need increased funding to extend their ability to cook fresh meals at school. One participant spoke about his frustration with what he considered to be a major barrier to implementing nutrition policies.

*…I would say number one’d be, financial. I think if…our school was to receive more money…a wider option of foods [would be served] instead of… just [using] the same distributors…bringing food that has been [cooked], they just need to heat up…[bring] fresh ingredients so that the cooks can cook something…most administrators…in the district care for their kids, but we need to sustain the school fiscally. (Participant #6, Elementary School Counselor).*

Some participants also mentioned limited autonomy to spend the state funds for nutrition-related purchases. Schools need to follow a bidding process, which restricts their choice to purchase from the distributor with the lowest bid. When asked to elaborate, one participant explained this process and also mentioned the need to relax this “state funding” restriction and place the “control” in the hands of the local schools to access high quality, fresh foods from local produce distributors. 

Physical activity was equally noted as being at the bottom of the “totem pole,” after reading, science, and math. Participants noted that “sports” get slightly more attention than PA but emphasized that not all children play sports.

#### Lack of strong leadership at the school district

In discussions of barriers surrounding nutrition policies, many participants immediately mentioned the lack of strong leadership from the district and the nutrition director. Participants mentioned that vending machines selling unhealthy snacks with “high fats, high calories, and high sugar” prevail on middle and high school campuses because school leadership values the revenue.

### Facilitators of policy implementation

#### Strong leadership at the school district

On the other hand, some participants praised the leadership from the school nutrition director who was seen as knowledgeable, creative, and committed, generating menus that the students enjoy while still adhering to state nutritional guidelines. One participant could not contain his enthusiasm when discussing the leadership exerted by their district nutrition director.

*…we have an-an excellent food service director who really…takes it to heart. She’s not only thinking about what the state wants to provide but she thinks about who she’s providing it for and so…when she chooses the foods that are available because…the food is already preset and simply you’re looking at a catalog…she chooses those foods that she is…thinking that our students [will] consume. (Participant #3, Family Mediation Director).*

#### Healthy norms for families and community through school-community linkages

All participants mentioned the importance of creating school-community linkages so that PA and nutrition policies are embraced and enforced by the community and students are surrounded by healthy norms inside and outside of school. One participant talked about “unity” across children, parents, and community, with the school being the unifying organization. When interviewees were asked to elaborate on how these connections could be made, participants mentioned communicating to parents through school events, such as parent’s information night and large community gatherings like health fairs, to create “awareness” of the policies and generate a message of “take care” of each other. Others mentioned that while awareness was important, taking action was also critical. Some participants had specific ideas on taking action through a coordinated effort led by a “committee” of community volunteers. Some saw the committee getting involved in schools by volunteering in the cafeteria, at recess time, or during PE. Others saw them assisting by raising funds. A participant mentioned bigger advocacy and engaging with the chamber of commerce and the city council to provide more infrastructure for PA. This participant also summed up the need to coordinate school-community efforts to provide students with PA opportunities outside of school to continuously motivate healthy behaviors.

*Because if they learn it here in school and they go…home and there’s no place to implement it [meaning what children have learned], then it makes it difficult. We—our kids don’t have a place to go and do that activity after school. And…if we had more [opportunities for PA after school], I think more…kids would get into it…(Participant #8, Superintendent)*

#### Pooling community resources

Many participants shared the importance of aggregating community resources around PA and nutrition and making them available to children and families. Resources from the city’s parks and recreation program were mentioned as well as the Young Men’s Christian Association (YMCA) and Amateur Athletics Union (AAU). One participant shared that these resources could be helpful for the kids after school and during summer.

*Rec[reational] leagues…the city’s park and recreation program reaching out to the community’s youth, in collaboration with the school district…that would help them continue [being physically active] outside of the school day…summers and weekends and…evenings. (Participant #2, Transportation Director).*

Another participant mentioned “promotion of the AAU” in schools because this encouraged kids to register for the program while helping the community engage in sports. Several participants also suggested opening the school gym for public use in the afternoons and evenings as most people do not have access to an affordable gym. They believed that the community could form a team of volunteers to manage the gym, and the school would need to only provide the space.

When discussing resources for nutrition, some participants could not contain their enthusiasm for their “beautiful” agricultural community and the variety of seasonal fruits and vegetables that are abundant during harvest time. They mentioned the need to establish and build partnerships with local farm owners to access fruits and vegetables when the farmers decide to let the orchard go. One participant eloquently described this process.

*…We have so many, so many assets in our community. We have fresh fruit…we see orchards where the farmer doesn’t pick the orchards because it’s cheaper to just let them fall. …We went and picked apples in an orchard this past year because a farmer was gonna…let the whole orchard go. And, you know, here’s 20 acres of apples. Why aren’t…we allowing access for the food bank…or volunteers to go out and pick apples? And share the apples or the produce with people. And that’s one of the things you see so much waste which…could be put…to good use and you could be giving it to people. (Participant #15, School Board Member)*

One participant shared that pooling community resources meant the whole is greater than the sum of its parts, and more things could be accomplished when they are brought together.

*…There’s so many opportunities out there, it just, you know, sometimes, it takes pooling resources…with the school and parks and rec. And we, we’ve done that before in the past, so…I, I think it’s important that the entities don’t think they have to do everything by themselves…[like]building partnerships. (Participant #11, Parks and Recreation Director)*

## Discussion

This study examined the knowledge and perceptions of school nutrition and PA policies as well as barriers and facilitators to policy implementation among rural school stakeholders. Participants were overwhelmingly supportive of policies promoting PA and a healthy diet, even when lacking a specific understanding of said policies. Four factors were identified as barriers to policy implementation: viewing PA as a low priority, misuse of recess time, lack of strong leadership, and funding constraints. Importantly, three factors were also noted as facilitators of policy implementation: strong leadership, school-community linkages were highly endorsed as a means of pooling community resources to improve nutrition and PA among children. To our knowledge, this is the first study that examines stakeholder perceptions of both PA and nutrition policies in rural schools. Furthermore, participants in the current study represented a wide range of school-based positions, including school board members, school leadership, teaching staff, and nutrition program personnel, allowing for perspectives from both high- and lower-level staff.

While a majority of study participants were confident their school had some policy surrounding PA, only 10% (*n* = 2) of participants were able to describe the policy in detail. This knowledge gap may reflect varying levels of support for the policy and its implementation between various school stakeholders [20]. For example, lack of guidance, limited monitoring, and low levels of accountability have been identified as barriers to PA policy implementation, and such environments may foster poor policy recall [31, 32]. With respect to nutrition, most participants were able to accurately describe one or more nutrition policies currently implemented in their school. Despite variance in their ability to recall specific policies, participants agreed that both PA and nutrition policies had tremendous impact on both the short- and long-term health of their students and their families.

Low priority levels and not using recess time for PA emerged as some of the main barriers to implementing PA policies in schools. These findings are consistent with results from a recent systematic review [20] that identified environmental context and resources, social influences, and competing curriculum priorities as prominent factors affecting PA policy implementation in schools. In the current study, identified factors largely map onto these three domains. Specifically, the current theme of low priority for PA most closely relates to competing curriculum priorities such as PE being pushed aside in favor of academic subjects, but may also stem from social influences (e.g., principal and district support for PA). Not using recess times for PA has emerged as a barrier to school-based PA in the literature and is most closely related to the theme of environmental context and resources [20]. Additionally, in the current study, participants noted that students watched movies indoors during recess when the weather was inclement as no appropriate indoor space was available for PA. As rural schools may lack the infrastructure and tools to implement effective PA policy, policy makers should collaborate with school stakeholders to understand their capacity and provide adequate funding to facilitate implementation [33, 34].

For nutrition policies, lack of strong leadership and funding constraints were identified as key implementation barriers. These findings are consistent with a study that classified factors influencing school implementation of nutrition policies into five themes: 1) level of macro support, 2) financial pressures, 3) alignment of core school priorities, 4) development of a common purpose among stakeholders, and 5) recognition of school and community characteristics [19]. Lack of strong leadership most closely aligns with the level of macro support. Past studies have emphasized the importance of leadership clearly communicating [34] and providing adequate support/training [35] to local educators and administrators tasked with ground-level nutrition policy implementation. The barrier of constrained funding most clearly maps onto the theme of financial pressure [14]. Similar to previous studies of school personnel [35, 36], study participants consistently reported funding constraints and inflexible procurement policies as limiting the schools’ ability to offer a wider variety of fresh foods to students. Instead of advocating for an either/or approach, cultivating a culture of support for nutrition at the district, state, or national level may facilitate the allotment of funding to enact these policies. 

Participants were largely supportive of developing a reciprocally beneficial relationship with other sectors of the community and believed such actions would reinforce healthy norms for both children and adults. This theme of recognizing the importance of school, family, and community connections and the overarching context in which nutrition and PA behaviors occur is particularly critical for effective and sustainable school-based nutrition and PA policy interventions. Policies should be designed based on a community’s specific characteristics, resources, and constraints [19]. For example, participants expressed high levels of enthusiasm in forming partnerships with local farmers to bring low-cost fresh fruits and vegetables into the schools. Utilizing this and other unique aspects of the rural community could potentially enhance PA and nutrition policy implementation and, ultimately, reduce rates of child obesity. Future studies may want to examine whether implementation of strategies addressing the barriers and enhancing facilitators lead to success in rural school settings.

Study findings should be considered in light of the following limitations. The study results are derived from four rural agricultural communities in Washington State; thus, the unique sociodemographic characteristics of these schools (majority Hispanic and nearly 100% of student body participates in the free/reduced-price lunch program) and the geographic contexts in which they are located may limit the generalizability of findings. Additionally, data from this study were collected in 2014 and may not reflect more recent themes and challenges. However, modifying school class structures is notoriously difficult [37], and it is likely that many of the barriers identified in this study continue to persist.

## Conclusions

Schools provide a unique setting to promote healthy diet and PA behaviors in children and their families. While a number of policy interventions have been enacted over the past decade, limited research has investigated how these policies are perceived and implemented by school stakeholders, particularly in rural settings. Results from this study suggest that while knowledge of the specific school nutrition and PA policies may vary, support for such policies remains high among rural school stakeholders. Making PA a high priority, ensuring designated PA times are used appropriately, displaying strong leadership, and having dedicated funding for nutrition policies emerged as critical factors for successful policy implementation in the school setting. Developing linkages between schools and the community was also identified as a way to improve nutrition and PA among children in the community. Study findings can inform policy development and support strategies for policy implementation in rural school settings.

## Supplementary Information

Below is the link to the electronic supplementary material.

Additional file 1. Key informant interview guide

## Data Availability

The datasets generated and analyzed during the current study are not publicly available to protect the privacy of the study participation, but are available from the corresponding author on reasonable request.

## Abbreviations

STRIDE: Strategizing Together Rural Interventions for Diet and Exercise
PA: Physical Activity
US: United States
CDC: Centers for Disease Control and Prevention
PE: Physical Education
AAU: Amateur Athletics Union
