# Publisher Correction: Factors impacting implementation of nutrition and physical activity policies in rural schools

**DOI:** 10.1186/s12889-023-15401-8

**Published:** 2023-03-21

**Authors:** Caryn Ausenhus, Joshua M. Gold, Cynthia K. Perry, Andrea T. Kozak, Monica L. Wang, Sou Hyun Jang, Judy Leong, Edgar Rodriguez, Catherine Duggan, Linda K. Ko

**Affiliations:** 1 Public Health Sciences Division, Fred Hutchinson Cancer Center, 1100 Fairview Ave. N, M3-B232, 98109 Seattle, WA USA; 2 Department of Health Systems and Population Health, University of Washington, Seattle, USA; 3 School of Nursing, Oregon Health & Science University, 3455 SW US Veterans Hospital Rd., SN-ADM,, 97239 Portland, OR USA; 4 Department of Psychology, Oakland University, 654 Pioneer Drive, 48309 Rochester, MI USA; 5 Department of Community Health Sciences, School of Public Health, Boston University, 715 Albany Street, 02118 Boston, MA USA; 6 Department of Sociology, Korea University, 145 Anam-Ro, Anam-Dong, Seongbuk-Gu, Seoul, South Korea; 7 U.S. Department of Veterans Affairs, VA Puget Sound Healthcare System, 1660 S. Columbian Way, 98108 Seattle, WA USA

**BMC Public Health (2023) 23:308**

10.1186/s12889-023-15176-y

In the original publication of this article there was an error in an author name. The correct and incorrect information is listed below. The original article has been updated. The publisher apologizes for the inconvenience caused to the authors.

Incorrect

Andrea Kozak

Correct

Andrea T Kozak

