# Supplementary material for: Factors impacting implementation of nutrition and physical activity policies in rural schools
Source: BMC Public Health. 2023 Feb 10;23:308. doi: 10.1186/s12889-023-15176-y (PMC9921364; doi:10.1186/s12889-023-15176-y)
Supplement: Supplementary file 1 — Additional file 1. Key informant interview guide [file 12889_2023_15176_MOESM1_ESM.docx]

**Key Informant Interview Guide**

1. **GENERAL QUESTIONS**
2. What are your thoughts about obesity? What comes to your mind when you hear the word “obesity”?
3. What are your thoughts about physical activity? What comes to your mind when you hear the word “physical activity”?
4. What are your thoughts about healthy eating? What comes to your mind when you hear the word “healthy eating”?

**2. CURRENT SCHOOL POLICIES AROUND HEALTHY EATING AND PHYSICAL ACTIVITY**

**Physical Activity**

1. Can you tell me what policies are in your school around physical education?
2. How do you think having a school policy around physical education can impact on how kids exercise?
3. What makes it difficult for schools to implement policies around physical education?
4. What would make it easier to implement policies around physical education?
5. Can you think of activities that can facilitate implementation of school policies around physical education?
6. What kinds of support can the community provide to facilitate implementation of policies around physical education in schools?

**Healthy Eating**

1. Can you tell me what policies are in your school around food services?
2. How do you think having a school policy around food services can impact what kids eat?
3. What makes it difficult for schools to implement policies around food offerings?
4. What would make it easier to implement policies around food offerings?
5. Can you think of activities that can facilitate implementation of school policies around food services/offerings?
6. What kinds of support can the community provide to facilitate implementation of policies around food services in schools?

**3. SCHOOL-BASED PROGRAMS FOR STUDENTS**

**Physical Activity**

1. What kinds of activities are currently available in your school to encourage kids to be more physically active?
2. What kinds of activities or events would be most helpful to kids in terms of physical activity?

**Nutrition**

1. What kinds of activities are currently available in your school to encourage kids to eat healthy?
2. What kinds of activities or events would be most helpful to kids in terms of nutrition?
